# ProAD – A database of rotary ion-translocating ATPases in prokaryotic genomes

**DOI:** 10.3389/fmolb.2024.1471556

**Published:** 2025-01-03

**Authors:** A. V. Litvin, A. S. Lapashina, A. P. Ermidis, M.S. Gelfand, B. A. Feniouk

**Affiliations:** ^1^ Center for Molecular and Cellular Biology, Skolkovo Institute of Science and Technology, Moscow, Russia; ^2^ A.N.Belozersky Institute of Physico-Chemical Biology, Lomonosov Moscow State University, Moscow, Russia; ^3^ Winogradsky Institute of Microbiology, Federal Research Center of Biotechnology, Russian Academy of Sciences, Moscow, Russia; ^4^ Faculty of Bioengineering and Bioinformatics, Lomonosov Moscow State University, Moscow, Russia

**Keywords:** rotary ATPase, ATP-synthase, F-ATPase, A-ATPase, V-ATPase, N-ATPase, prokaryotic

## Introduction

Rotary ion-translocating ATPases (ATP synthases) are multi-subunit membrane enzymes that catalyze the synthesis of ATP from ADP and inorganic phosphate. The energy for ATP synthesis is provided by ion transport through the enzyme, driven by the transmembrane electrochemical ion potential difference. In many prokaryotes, the enzyme functions in reverse, pumping ions across the membrane using energy derived from the ATP hydrolysis; see (Kühlbrandt, 2019; Zubareva et al., 2020) for reviews. The ion specificity of rotary ATPases, defined by their membrane-embedded subunits, can be H^+^ or Na^+^.

Rotary ATPases are classified into three groups: F-ATPases found in many bacteria and eukaryotic organelles (mitochondria, chloroplasts), V-ATPases typical for plasma or vacuolar membranes of eukaryotic cells, and A-ATPases found in most archaea and some bacteria, which are structurally closer to V-type than to F-type enzymes (Ihara et al., 1992). F-, A-, and V-ATPases share the catalytic core (subunits involved in the ATP synthesis/hydrolysis and ion translocation) but have different auxiliary subunits. Some researchers also distinguish N-ATPases, a specific subclass of F-type enzymes first described by Dibrova et al. that forms a separate branch on the phylogenetic tree of rotary ATPases (Dibrova et al., 2010). The main function of N-ATPases is presumed to be not ATP synthesis, but ATP-driven ion pumping and ion gradients maintenance.

Rotary ion-translocating ATPases are believed to share a common evolutionary origin, as evidenced by their analogous structural features and catalytic mechanisms (Mulkidjanian et al., 2007). This implies that such enzyme was already present in the last universal common ancestor (LUCA) of bacteria, archaea, and eukaryotes (Gogarten and Taiz, 1992).

Rotary ATPase of pathogenic bacteria are promising targets for development of new antibiotics (Hards and Cook, 2018). Bedaquiline, a diarylquinoline that binds to the membrane domain of *Mycobacterium tuberculosis* ATP synthase and blocks proton translocation through the enzyme (Koul et al., 2007), is used to treat multidrug-resistant tuberculosis (Pym et al., 2016). Understanding the variety and distinctive features of bacterial rotary ATPases is an important step in developing new antimicrobials that selectively target the enzyme in pathogens without affecting the mitochondrial ATP synthase in human cells.

We analyzed genomes of bacteria and archaea from the Genome Taxonomy Database (GTDB) v202 to find genes encoding rotary F-, A-, and N-ATPases.

We present the Prokaryotic Rotary ATPase Database (PRoAD) that contains structures of ATPase genomic loci and predictions for ion specificity of rotary ATPases. Most prokaryotic genomes encode only a single rotary ATPase, but some have genes for multiple ATPases with different or the same predicted ion specificities, whereas some prokaryotes lack genes encoding rotary ATPases.

## Methods

### Identification of genes encoding rotary ATPases in GTDB genomes

To identify rotary ATPase subunits, we created HMM-profiles (hmmbuild, HMMER 3.3.2) for subunits of F/N-ATPases (αβγε*abc*) and F-ATPase-specific δ, as well as A-ATPase subunits (ABDEFGI) (Table 1). These profiles were based on genes from 713 genomes in the COG database (Tatusov et al., 2001; Galperin et al., 2019). The HMM-profile for A-ATPase subunit K was constructed using the alignment from Mulkidjanian et al. (2008). The HMM profiles were used to search against translated coding sequences from GTDB v202 (hmmsearch, HMMER 3.3.2), with hits filtered by manually selected bitscore cutoffs (see Supplementary Material).

**TABLE 1:** Subunits of prokaryotic rotary ATPases, their evolutionary relationship and function.

Subunit name		Function
F/N-ATPase	A-ATPase	
**α**	**B**	Catalytic, carry nucleotide-binding sites
**β**	**A**	
**γ**	**D**	Connect cytoplasmic and membrane parts of the enzyme, provide the coupling between ATP synthesis/hydrolysis and transmembrane ion transport
δ**	-	
ε	F*	
***a***	**I**	Mediates transmembrane ion transport, forms two ion-permeable half-channels in the membrane
b**	E*, G*	Connects the cytoplasmic and membrane parts of the enzyme
***c***	**K**	Mediates transmembrane ion transport, carries the ion-binding site

Subunits placed in the same table line are thought to be orthologous. For subunits marked *, the orthology is debatable (Zubareva et al., 2020). Subunits highlighted in bold represent the catalytic core. ** In N-ATPases, subunit δ is absent or fused with subunit *b*.

We grouped the ATPase subunits into sets encoding functional enzymes, assuming that genes encoding subunits of the same ATPase are colocalized (Koumandou and Kossida, 2014). For each organism we determined the tentative, global operon structure of the genome (see Supplementary Materials for details). All coding DNA sequences (CDSs) were grouped into candidate operons based on the following criteria: (1) CDSs are located on the same DNA strand consecutively, and (2) the distance between adjacent CDSs is less than 200 nucleotides. Among the candidate operons, we selected those containing at least one rotary ATPase subunit gene and examined their relative positions. If the distance between two such operons was less than 1,000 nt, they were clustered together. Gene clusters containing complete sets of subunits (nine for A-ATPases, eight for F-ATPases, and seven for N-ATPases; see below) were considered to encode functional enzymes. The remaining clusters were combined, when possible, to form complete sets, which were also regarded as individual enzymes.

N-type ATPases with fused subunits *b* and δ were identified as encoded by a single gene cluster with a specific gene order (βεXX*ac* (*b*δ)αγ, where “(*b*δ)” indicates that hmm-search hits for *b* and/or δ that are encoded by one CDS, and two specific to N-ATPase genes (X) that lie between ε and *a* subunits). We also mapped all identified N-ATPases onto phylogenetic trees based on the multiple sequence alignment of α and β subunits. As expected, most N-ATPases formed a distinct clade on these trees. However, this clade also contained several enzymes annotated as F-type. Furthermore, several ATPases assigned to the N-type were found outside this clade. These cases were re-annotated individually after manual inspection of their gene cluster structure and their positions on the phylogenetic trees.

Some genomes were found to contain exact copies of ATPase gene clusters. We considered such repeats as resulting from genome misassembly and retained only one copy.

In many genomes, this procedure yielded incomplete sets of rotary ATPase subunits genes. Generally, subunits not directly involved in the catalysis (e.g., subunit *b* in F-type ATPase) are less conserved than the catalytic core of the enzyme. These subunits were more challenging to identify and could be encoded in a given genome but remain undetected by our approach. Since prokaryotic genomes typically contain very few nonfunctional sequences (Lawrence et al., 2001; Koonin, 2009), we assumed that a genome encoded a fully functional rotary ATPase if it contained the genes of the essential catalytic subunits (αβγ*ac* for F- or N-type ATPase and ABDIK for A-ATPase).

A considerable number of genomes did not encode the complete set of essential subunits. One possible explanation, particularly for genomes assembled from metagenomic data, was genome incompleteness. In the GTDB phylogenetic tree, low-completeness genomes lacking complete rotary ATPases most often clustered with more complete genomes that contained all genes for essential rotary ATPase subunits. Therefore, to describe the phylogenetic distribution of rotary ATPase combinations, we grouped the genomes by genus and selected the most representative (i.e., the most frequent) combination for each genus. Nonetheless, we observed entire genera with many species lacking rotary ATPase genes. These cases are discussed below in the Data Review section.

### Prediction of the ion specificity

Ion specificity for each rotary ATPase was predicted based on the sequence of its ion-binding subunit (*c* in F- and N-ATPases, K in A-ATPases). The simplest *c*/K subunit is a hairpin comprising two transmembrane α-helices bearing a single Glu or Asp residue located in the middle of the C-terminal helix, which binds H^+^ or Na^+^. More complex *c*/K variants consist of two or more hairpin domains, each of which may or may not contain an ion-binding site.

We grouped and aligned the subunits with similar numbers of hairpins. In the resulting alignments, we manually searched for the ion-binding residues (Glu/Asp). If the ion-binding site contained the complete Na^+^-motif described in Mulkidjanian et al. (2008), we considered it Na^+^-specific. If at least one Na^+^-specific residue was missing, the site was considered H^+^-specific. Finally, if a multiple-hairpin subunit contained a combination of H^+^- and Na^+^-binding sites, it was annotated as Na^+^-specific (See Supplementary Materials for details).

### Data review

The data are organized in a table, where each row represents an ATPase subunit gene (see Supplementary Materials for details). Our analysis included genomes of 45,555 bacteria (12,037 genera) and 2,339 archaea (851 genera). In total, we found 48,143 rotary ATPases containing all the core subunits, specifically, 37,169 F-type (35,086 H^+^-specific, 2063 Na^+^-specific), 1807 N-type (893 H^+^-specific, 914 Na^+^-specific), and 9167 A-type (4199 H^+^-specific, 4,927 Na^+^-specific). Surprisingly, 20 candidate F/N-ATPases and 41 A-ATPases had *c*/K subunits without essential ion-binding Glu/Asp residues. These proteins were marked as “non-catalytic”.

### Phylogenetic distribution of rotary ATPases

**FIGURE 1:**
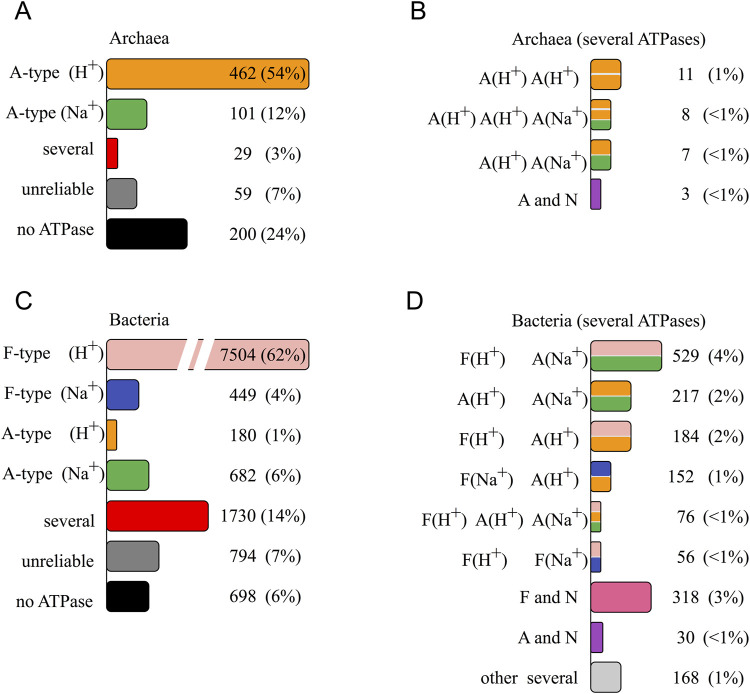
Distribution of rotary ATPases encoded in prokaryotic genomes from GTDB database v202 by genera. The most frequently occurring combination was taken for each genus. Numbers of genera and percentage are given for each combination. Red columns in **(A, C)** (marked “several”) depict genera where several ATPases are encoded in genomes of most species, and detailed distribution for them is given in **(B, D, A)** archaeal genomes; **(C, D)** bacterial genomes. See details in text.

Most archaeal genomes harbor a single A-ATPase, which translocates either H^+^ or Na^+^ (Figure 1A). In 3% genera, we observed a combination of two or more A-ATPases (red column “several” in Figure 1A). The remaining 31% of archaeal genera in GTDB database lacked the complete set of core ATPase subunit genes.

In seven archaeal genera we found two A-ATPases with different predicted ion specificity (Figure 1B). We also identified taxa in which two H^+^-translocating A-ATPases coexisted within a single genome (11 genera). Furthermore, in eight genera of the order Methanomicrobiales (phylum Halobacteriota), the prevalent combination was two H^+^-A-ATPases and a Na^+^-A-ATPase.

N-ATPases were rare in archaea. In genus *Methanosarcina* (phylum Halobacteriota, class Methanosarcinia) we found a combination of H^+^-A-ATPase and H^+^-N-ATPase in 17 of its 25 species. Additionally, we found six genomes in other genera of the same class that contain N-ATPases (either H^+^- or Na^+^-translocating) together with a H^+^-A-ATPase. A unique case is a genome from the genus *Methanothrix_A*, harboring two A-ATPases (one H^+^- and one Na^+^-translocating) and a single Na^+^-translocating N-ATPase.

F-ATPases appear to be absent in archaea. We identified several genes potentially encoding F-ATPase subunits in six archaeal genomes that lack the complete set of essential genes, suggesting that they most likely do not encode functional enzymes.

The distribution of rotary ATPases in bacterial genomes was more complex (Figure 1C). Most bacteria contain a single F-ATPase, translocating either H^+^ or Na^+^ (62% and 4% of genera, respectively). In 7% of the genera we found a single A-ATPase, which was Na^+^-translocating in 682 and H^+^-translocating in 180 genera.

The presence of multiple rotary ATPases of different types was rather common in bacterial genomes (Figure 1D). In 3% genera a N-ATPase was found alongside a F-ATPase. The most frequent combination was a H^+^-translocating F-ATPase with a Na^+^-translocating N-ATPase, found in 164 genera. However, there were species where both enzymes were predicted as H^+^-translocating, found in 136 genera. Some genomes encoded combinations of Na^+^-F-type enzymes with N-ATPases. A combination of Na^+^-F-ATPase and H^+^-N-ATPase was found in only one genus (DSBG01 sp011046135, Desulfobacterota). Both Na^+^-dependent F-type and N-type ATPases were found in genomes of six genera belonging to Firmicutes_E, A, F. Nine genera featured H^+^-F-ATPase and two N-ATPases. In phyla Chloroflexota, Chlamydiota, a N-ATPase is found alongside an A-ATPase. Here, all combinations of ion specificity types can be observed: Na^+^-A+ Na^+^-N (seven genera), H^+^-A+ Na^+^-N (nine genera), Na^+^-A+ H^+^-N (five genera), and H^+^-A+ H^+^-N (five genera). In three genera, genomes encode two A-type and one N-type rotary ATPases. Genus *Amnivibrio* includes two genomes with three A-ATPases and one N-ATPase encoded.

N-type ATPase is typically found together with F- or A-type ATPases in bacterial genomes (Dibrova et al., 2010). However, in genus *T78* (phylum Chloroflexota) the majority of genomes (18 out of 20) encode a single rotary ATPase of H^+^-N-type, and only two species also contain A-ATPase subunits genes.

A widespread case in bacterial genomes is a combination of several F- or A-ATPases (Figure 1D). We found genomes encoding up to five rotary ATPases of different types. Most of these genomes encode two to three rotary ATPases: one F- and one A-ATPase in 890 genera, two A-ATPases in 222 genera, one F- and two A-ATPases in 103 genera, two F-ATPases in 78 genera, and, finally, combined F-, A-, and N-ATPase in 42 genera. Typically, if a genome encodes several rotary ATPases, they differ in the ion specificity, i.e., an H^+^-ATPase comes together with a Na^+^-ATPase (1,137 genera of 1,388). Nevertheless, we have found 220 genera with two F/A-ATPases both predicted as H^+^ and 23 genera with two F/A-ATPases both predicted as Na^+^.

We did not find genes for rotary ATPase core subunits in genomes of 6% bacterial and 24% archaeal genera (Figures 1A, C). At that, among 2,969 genomes with no ATPase genes, 1,567 genomes belonged to genera in which some genomes contained ATPase genes. This can be explained by genome incompleteness, especially in many species of Patescibacteria, a superphylum predominantly comprising organisms identified through metagenomic and single-cell sequencing of uncultivated species.

However, in several phylogenetic groups (large genera, families, orders), a significant fraction of genomes lack ATPase genes. Genome incompleteness is not sufficient to explain these observations. Prokaryotes that appear to lack rotary ATPase include:

(i) Phytoplasmas, which are obligate intracellular plant parasites. The first sequenced phytoplasma lacked rotary ATPase (Bai et al., 2006). Previously, rotary ATPases were thought to be indispensable for cellular life, and their genes were included in the proposed minimum gene set for the cellular existence (Mushegian and Koonin, 1996).
(ii) Insect endosymbionts, e.g., *Mikella*, *Riesia*, *Nardonella*, *Tremblaya*, *Hoaglandella*, *Gullanella* of Proteobacteria. Genomes are notably reduced in endosymbionts (Latorre and Manzano-Marín, 2017), and, in some cases, have lost rotary ATPase genes.
(iii) Components of the gut microbiota, e.g., o__TANB77 (Firmicutes_A, class Clostridia), f__CAG-508 (Firmicutes_A), f__UBA660 (Firmicutes, class Bacilli).
(iv) Archaea from taxa: p__Aenigmatarchaeota, p__Huberarchaeota, p__Nanoarchaeota (including o__Pacearchaeales), p__Iainarchaeota, and o__B26-1 and o__EX4484-135 (within p__Thermoproteota, class c__Bathyarchaeia).

Several groups, including DPANN archaea (Aenigmarchaeota, Pacearchaeota), Bathyarchaeia and Patescibacteria, have been reported to lack ATPase genes (Mahendrarajah et al., 2023). To our knowledge, the physiology of prokaryotes lacking rotary ATPase genes that are neither endosymbionts nor parasites, has not been studied. These organisms might rely on fermentation processes and make ATP through substrate-level phosphorylation. It is not clear, however, how they generate the transmembrane electrochemical ion potential difference and maintain the pH homeostasis.

## Data Availability

Publicly available datasets were analyzed in this study. This data can be found here: https://gtdb.ecogenomic.org/. The ProAD adatabase described in this study can be found in the Github repository: https://github.com/ATP-synthase-lab/Prokaryotic_Rotary_ATPase_Database.
