# Supplementary material for: ProAD – A database of rotary ion-translocating ATPases in prokaryotic genomes
Source: Front Mol Biosci. 2025 Jan 3;11:1471556. doi: 10.3389/fmolb.2024.1471556 (PMC11738941; doi:10.3389/fmolb.2024.1471556)
Supplement: Supplementary file 1 [file DataSheet1.pdf]

Supplementary material for

"ProAD: A Database of Rotary Ion-Translocating ATPases in Prokaryotic Genomes"

Workflow for Database construction

For each prokaryotic genome from the GTDB database we first clustered the protein-coding open reading frames into candidate operons (Fig 1S, A). Two protein-coding open reading frames were considered to belong to the same candidate operon if they were located on the same DNA strand consecutively, and if the distance between them was less than 200 nucleotides.

The next step was to allocate the open reading frames coding rotary ATPases subunits. Hmm-profiles for the search of ATPase subunits were made using sequences of subunits from COG database. Subunits were aligned with muscle v5.1 with default parameters and manually adjusted. Poorly aligned regions were removed manually, hmm-profiles were built with hmmbuild, package HMMER 3.3.2.

If two open reading frames coding rotary ATPase subunits were found by hmmsearch in the same candidate operon, they were considered to belong to the same operon. If two operons coding rotary ATPase subunits were less than 1000 nucleotides apart, they were merged into a single gene cluster, even when located on different DNA strands (Fig. 1S, B).

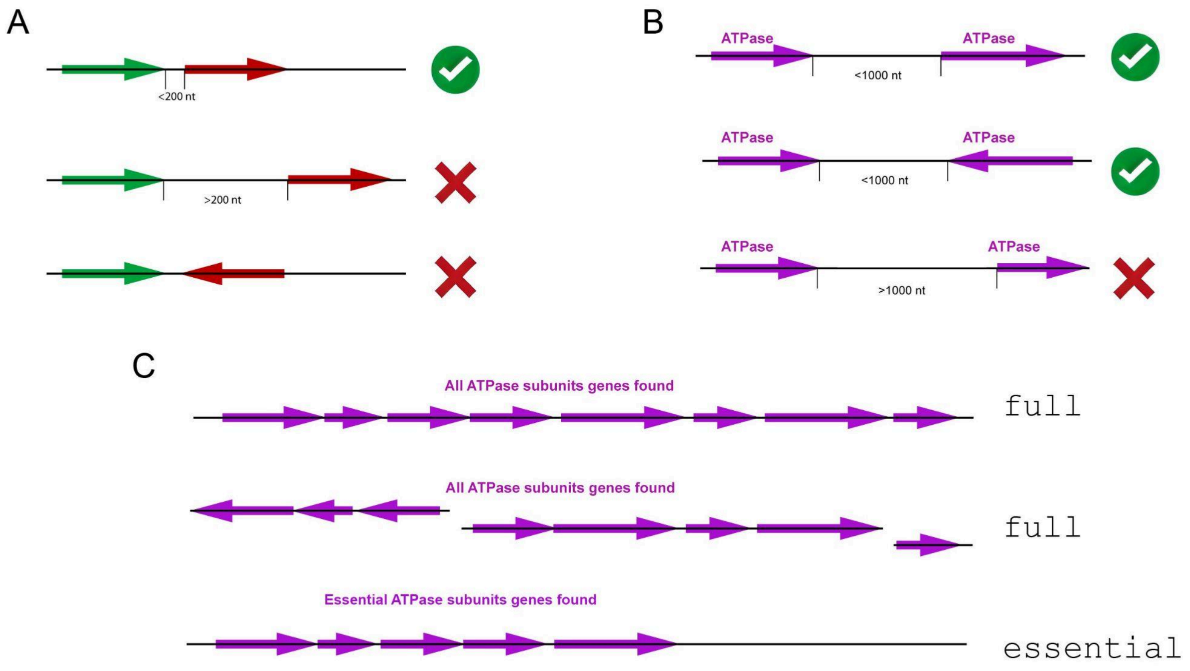

Fig. S1 Scheme of the workflow for ProAD database construction.

Finally, gene clusters containing complete sets of subunits (ABCDEFGIK for A-ATPases, αβγδε*abc* for F-ATPases, and αβγ[δ*b*]ε*ac* for N-ATPases) were marked in the database as "`full`". Most often it was a single operon, but in many organisms it was two or more gene clusters coding rotary ATPase subunits (Fig. 1S C).

When it was impossible to make a complete set of subunits (genes of some subunits were not found), we assumed that a genome encodes a fully functional rotary ATPase if it contains the genes of all the essential catalytic subunits (αβγ*ac* for F- or N-type ATPases and ABDIK for A-ATPases). Such cases were marked as "`essential`" in the database.
Gene clusters with incomplete subunit sets where some of the essential subunits were missing were also defined as probable "enzymes" and assigned a unique identifier, but were not used in the analysis of rotary ATPase distribution among prokaryotes.

Prokaryotic Rotary ATPase Database structure.

1. **Subunits of rotary ATPases**

The HMM profiles used to identify rotary ATPase subunits are in folders `hmms_FN` and `hmms_A` (for F/N-type enzymes and for A-type enzymes, respectively).
The data are organized in a table, where each row represents an ATPase subunit gene. Columns describe the gene's source (genome accession number, species, DNA strand, genomic coordinates) and the enzyme to which the subunit is assigned (unique enzyme ID, ATPase type, ion specificity, subunit composition, and gene cluster structure), etc.

Files `ATPases_subunits_A.csv.gz` and `ATPases_subunits_FN.csv.gz` contain the information on individual subunits of rotary ATPases. For each subunit the following information is present:

`accession` - genome accession number (Submitted GenBank/Refseq assembly)

`species` - species name

`seq_id` - GenBank genome sequence id

`fasta_id` - coding sequence unique identifier

`N` - number of the gene on the contig

`ID` - identifier of the protein in the genome

`start` - start position for the open reading frame

`end` - end position for the open reading frame

`strand` - DNA strand

`subunit` - subunit full name (not unique for the gene in case of fusions).

`fusion` - when two subunits are fused into one, two lines appear in the table with the same ID, and the fused subunits are identified in this column. For F- and N-type ATPases (file `ATPases_subunits_FN.csv.gz`) subunits α, β, γ, δ and ε are indicated as A, B, C, D, and E, respectively. E.g., "AD" for an F-ATPase denotes the αδ fusion.

`bitscore` - bitscore of the HMM search

`evalue` - e-value of the HMM search

`ion` - ion specificity of the c/K subunits predicted from the sequence. If at least one position of the Na^+-binding motif did not contain a Na^+-specific residue, the site was considered as H^+-specific.

`gcid` - unique identifier of the gene cluster

`gcst_full` - order of the subunit genes in the gene cluster. For F- and N-type ATPases (file `ATPases_subunits_FN.csv.gz`) subunits α, β, γ, δ and ε are indicated as A, B, C, D, and E, respectively. Fusions are marked with brackets: e.g. (AD) for an F-ATPase denotes the αδ fusion. Genes in a gene cluster that are coding proteins that are not rotary ATPase subunits are marked as "_".

`enz` - unique enzyme identifier in the Prokaryotic Rotary ATPase Database

`enz_ion` - ion specificity of the enzyme predicted from the structure of the *c*/K subunit. If the conserved Glu/Asp residue involved in the ion translocation is absent in the sequence, it is marked as "nc" (non-catalytic). If no gene for the *c*/K subunit has been found for the enzyme, it is marked as "-"

`enzst_full` - genome organization of the ATPase genes. If subunits are encoded by two or more gene clusters, these clusters are joined with a "+" sign. Gene designation is the same as for `gcst_full` above.

`FAN` - type of rotary ATPase (F, A, or N)

`essential` - states whether all genes encoding core catalytic subunits were found in the genome (α, β, γ, *a* and *c* for F-type; (αδ), β, γ, *a* and *c* for N-type; A, B, D, I, and K for A-type)

`full` - states whether all genes encoding the ATPase subunits were found in the genome (α, β, γ, δ, ε, *a, b* and *c* for F-type; (αδ), β, γ, δ, ε, *a, b* and *c* for N-type, ABCDEFGIK for A-type)

2. Rotary ATPases in prokaryotes

File `ATPases_by_species.csv` contains the information on individual rotary ATPases found in bacterial and archeal genomes from the GTDB database. For each rotary ATPase the following information is provided:

`accession` - genome accession number (Submitted GenBank assembly)

`species` - species name

`enz` - unique enzyme identifier in the Prokaryotic Rotary ATPase Database

`enz_ion` - ion specificity of the enzyme predicted from the structure of the *c*/K subunit. If the conserved Glu/Asp residue involved in the ion translocation is absent in the sequence, it is marked as "nc" (non-catalytic). If no gene for the *c*/K subunit has been found for the enzyme, it is marked as "-"

`enzst_full` - genome organization of the ATPase genes. If subunits are encoded by two or more gene clusters, these clusters are joined with a "+" sign. For F- and N-type ATPases subunits α, β, γ, δ and ε are indicated as A, B, C, D, and E, respectively. Fusions are marked with brackets: e.g. (AD) for an F-ATPase denotes the αδ fusion.

`FAN` - type of rotary ATPase (F, A, or N)

`essential` - states whether all genes encoding core catalytic subunits were found in the genome (α, β, γ, *a* and *c* for F-type; (αδ), β, γ, *a* and *c* for N-type; A, B, D, I, and K for A-type)

`full` - states whether all genes encoding ATPase subunits were found in the genome (α, β, γ, δ, ε, *a, b* and *c* for F-type; (αδ), β, γ, δ, ε, *a, b* and *c* for N-type, ABCDEFGIK for A-type)

`d`, `p`, `c`, `o`, `f`, `g` and `s` - taxonomy of the organism (domain, phylum, class, order, family, genus and species)

`genome_completeness, genome_contamination` — genome parameters from GTDB database.

3. Visualization of rotary ATPases types in the phylogenetic trees of bacteria and archaea.

We took phylogenetic trees from the GTDB r202 database and mapped the types of rotary ATPases on them (folder `phylo_distribution_pdf`). Each leaf in the tree represents a family. Each genus in a family is shown as a circle colored according to the type and number of rotary ATPases encoded in the genome of representative species.

The bacterial tree is splitted into several parts. Ten big phyla are shown separately. Other tree parts are named according to the phyla. The most comprehensive bacterial tree part is `Other_bacteria.pdf` that includes notes on collapsed branches that are shown separately.

Color codes used in the trees:

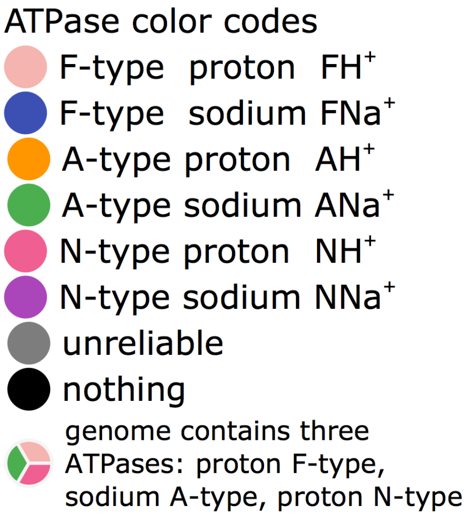

File `ATPases_representative_by_genera.csv` contains the information used to map the types and numbers of rotary ATPases on the phylogenetic trees from the GTDB r202 database.

4. Predicting the ion specificity of the prokaryotic rotary ATPases.

The comparison of the structures of the membrane-embedded oligomeric *c*/K-subunit rings of sodium-dependent F- and A-ATPases published by Mulkidjanian et al. in 2008 (The past and present of sodium energetics: may the sodium-motive force be with you. *Biochim. Biophys. Acta* 1777, 985–992) revealed nearly identical sets of amino acids involved in sodium binding. Several structures of *c*/K rings resolved for sodium-specific F- and A-ATPases confirm that Na^+ ion possesses a coordination number of six, delivered by specific amino acid ligands on subunits *c*/K that maintain it within the non-polar interior of the membrane. Here, to confirm the presence of these amino acids, all *c*/K subunit sequences were first grouped according to the number of transmembrane α-helical hairpins. Sequences in each group were aligned (muscle v5.1 default mode), and the alignments were manually corrected. Columns corresponding to the specific proton- and/or sodium-binding residues were determined manually in each alignment and each hairpin. Any hairpin was considered to be ion-binding if it carried [E/D] residue in the middle of the second transmembrane α-helix. A single-hairpin sequence was considered to be sodium-binding if it carried the motif [Q/E]-X_n-[E]-[S/T]-[S/T/Q]-X-X-[Y] (Fig. S2 left, and Fig. S3, A).
For multi-hairpin sequences we applied special rules of the residue location: the hairpin was considered sodium-binding if it contained [Q/E]-X_n-[E] motif and the previous hairpin contained [S/T]-[S/T/Q]-X-X-[Y] in its second α-helix (Fig. S2 right, and Fig. S3, B-D).

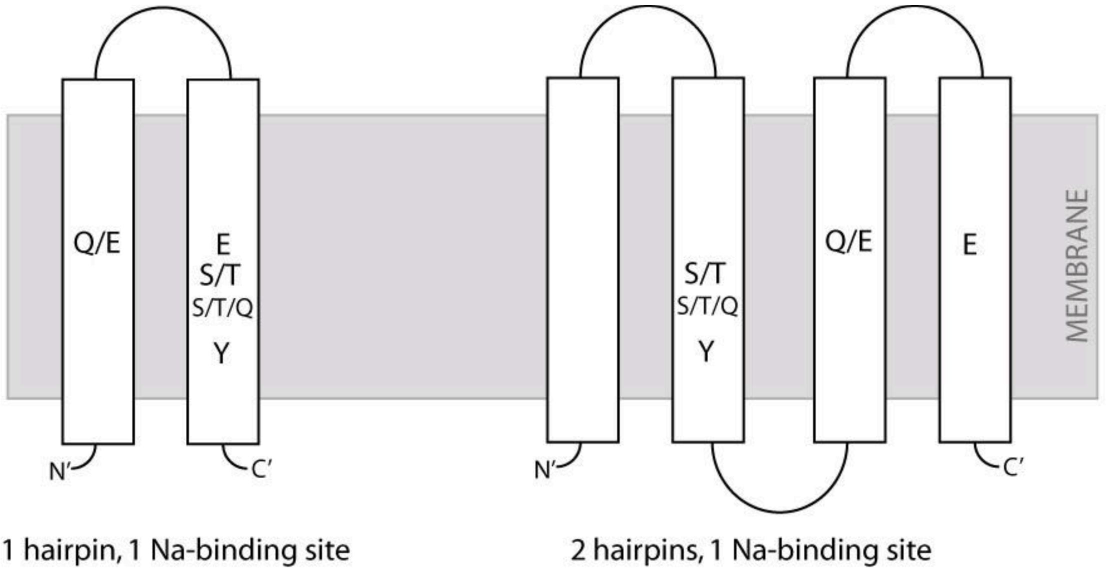

Figure S2. Sodium-binding amino acid residues in c/K subunits of rotary ATPases. Left - *c*/K subunit with 1 hairpin, right - *c*/K subunit with 2 hairpins.

Lack of even one of Na^+-binding groups is expected to significantly decrease the binding affinity. Therefore we considered the enzyme as Na^+-specific only when all the sodium-specific residues were present. Multi-hairpin sequences were assigned sodium-specific if they contained at least one sodium-binding site. The logos for multiple alignments of *c* and K subunits are given below in Fig. S3.

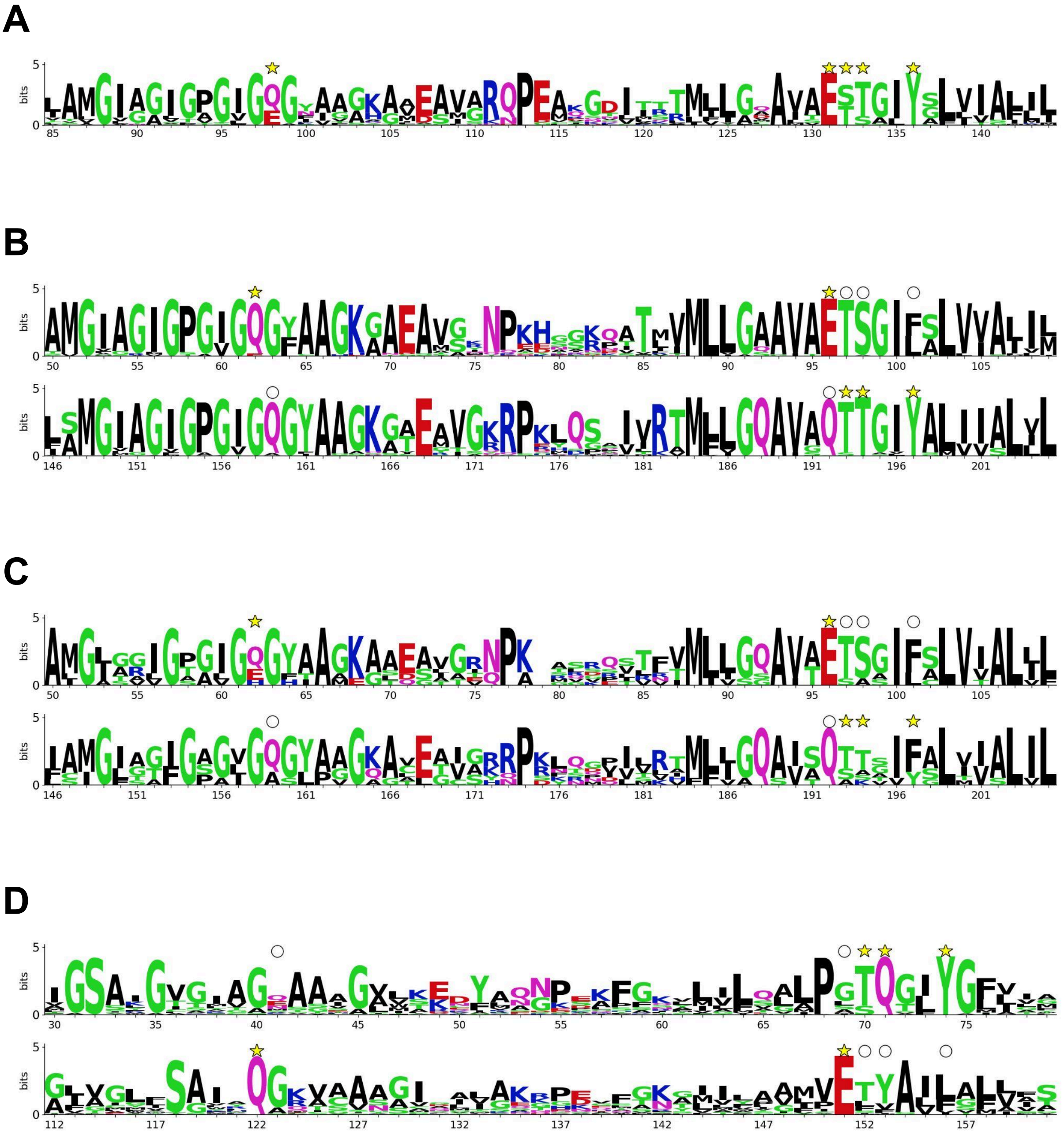

Figure S3. The logos for multiple alignments of rotary ATPase *c* and K subunits. A - *c*-subunit with 1 hairpin; B - *c*-subunit with 2 hairpins, one H^+-binding site and one Na^+-binding site; C - *c*-subunit with 2 hairpins, only one 1 Na^+-binding site; D - K-subunit with 2 hairpins, only one 1 Na^+-binding site. Yellow stars mark residues involved in Na^+-binding, hollow circles are positions where the Na^+-binding residues were supposed to be but are missing.

Supplementary Table 1

Bitscore cutoffs that were manually selected for the identification of rotary ATPases subunits by HMM-profiles in GTDB genomes.

F/N subunit	bitscore cutoff		A-subunit	bitscore cutoff
α	260		A	250
β	260		B	250
γ	50		C	40
δ	15		D	40
ε	30		E	50
a	30		F	20
b	30		G	40
c	10		I	50
			K	10
